# Screening for developmental disabilities in HIV positive and HIV negative children in South Africa: Results from the Asenze Study

**DOI:** 10.1371/journal.pone.0199860

**Published:** 2018-07-03

**Authors:** Justin Knox, Stephen M. Arpadi, Shuaib Kauchali, Murray Craib, Jane D. Kvalsvig, Myra Taylor, Fatimatou Bah, Claude Mellins, Leslie L. Davidson

**Affiliations:** 1 Department of Epidemiology, Mailman School of Public Health, Columbia University, New York, NY, United States of America; 2 Department of Pediatrics, College of Physicians and Surgeons, Columbia University, New York, NY, United States of America; 3 Department of Paediatrics and Child Health, Nelson R Mandela School of Medicine, University of KwaZulu Natal, Durban, South Africa; 4 Department of Public Health Medicine, University of KwaZulu-Natal, KwaZulu Natal, South Africa; 5 HIV Center for Clinical and Behavioral Studies, New York State Psychiatric Institute, New York, NY, United States of America; 6 Department of Sociomedical Sciences, Mailman School of Public Health, Columbia University, New York, NY, United States of America; The Ohio State University, UNITED STATES

## Abstract

**Background:**

While neurodevelopmental abnormalities are common in children with HIV infection, their detection can be challenging in settings with limited availability of health professionals. The aim of this study was to assess the ability to identify developmental disability among HIV positive and HIV negative children living in South Africa with an internationally used screen.

**Methods and findings:**

This analysis uses a sample of 1,330 4–6 year old children and 1,231 of their caregivers in KwaZulu-Natal, South Africa, including administration of the Ten Questions (TQ) screen, a standardized medical history and physical examination conducted by a medical doctor, with hearing and vision screening, psychological assessment for cognition and language delay, and voluntary HIV testing. There was a high prevalence of disability among the sample. Compared to HIV negative children, HIV positive children were more likely to screen positive on at least one TQ item (59.3 vs 42.8%, *p* = 0.01), be delayed in sitting, standing or walking (OR 3.89, 95% CI = 2.1–7.2) and have difficulty walking or weakness in the arms or legs (OR = 2.7, 95%CI = 0.8–9.37). By medical doctor assessment, HIV positive children were more likely to be diagnosed with gross motor disability (OR = 3.5, 95%CI = 1.3–9.2) and hearing disability (OR = 2.5, 95%CI = 1.2–5.3). By independent psychological assessment, HIV positive children were more likely to have cognitive delay (OR = 2.2, 95%CI = 1.2–3.9) and language delay (OR = 4.3, 95%CI = 2.2–8.4). Among HIV positive children, the sensitivity and specificity of the TQ for serious disability (vs. no disability) was 100% and 51.2%, respectively. Among HIV-negative children, the sensitivity and specificity of the TQ for serious disability (vs. no disability) was 90.2% and 63.9%, respectively.

**Conclusions:**

In this first report of the use of the TQ screen in the isiZulu language, it was found to have high sensitivity for detecting serious developmental disabilities in children, especially HIV positive children. The performance of the TQ in this sample indicates utility for making best use of limited neurodevelopmental resources by screening HIV positive children.

## Introduction

Neurodevelopmental disabilities, including impaired brain growth, motor, cognitive and language development, were among the earliest recognized features of pediatric HIV infection, affecting as many as 50% of children prior to availability of effect antiretroviral therapies (ART) [1–3]. Although early initiation of ART appears to prevent many of the most severe sequelae, neurologic impairment remains an important co-morbidity among children living with HIV [4–7]. Insults to the brain from HIV and associated illnesses during early child development may impede optimal social, emotional, physical, and educational functioning and outcomes resulting in impairments, limitations and restrictions that persist throughout childhood and adolescence and beyond.

Increased availability of ART in low- and middle- income countries, the home for more than 90% of HIV positive children, has resulted in great improvements in survival. As a result, the burden and character of neurodevelopmental disabilities throughout childhood has emerged as an important area for research, clinical care, policy and planning for health, educational and social services sectors in many high burden countries [8–10].

In general, the capacity for evaluating neurodevelopment of children living in many low- and middle- income countries is poor due to limited numbers of specialist healthcare professionals. A 2-stage strategy involving an initial screening questionnaire followed by more extended evaluation for those identified at-risk has been devised to overcome human resource shortcomings. The Ten Question (TQ) is a widely used screen that measures caregiver perception of how well their child functions compared to his or her peers in the realms of neurodevelopmental functioning. It was developed to identify serious (moderate and severe) cognitive, motor, seizure, speech, vision and hearing disabilities and developmental delays in settings with limited access to professional resources [11]. It can also be used as a planning tool for educators, health professionals and social services. The TQ has been translated into over 25 languages and was used by UNICEF in the Multiple Indicator Cluster Survey program as a module to estimate the prevalence of disability in children [12]. The TQ demonstrated good validity and reliability to identify children with serious disabilities. The validation, using known psychological measures and a standardized assessment of disabilities by a biomedical doctor, was carried out on approximately 22,000 children in 3 cultures including Jamaica, Bangladesh, and Pakistan [13]. However, the validity and potential utility of the TQ for assessing HIV positive children for risk of neurodevelopmental disabilities is not known.

South Africa, a middle-income country, has among the highest HIV/AIDS prevalence in the world with 7.1 million people living with HIV in 2016, including 320,000 children (ages 0–14) [14]. Universal access to ART throughout its public health system began in 2004 and has allowed approximately three quarters of HIV+ adults and children to receive ART [15], compared to an estimated 49% of children worldwide [16]. Because of the limited availability of trained medical doctors; there are 0.8 physicians per 1,000 persons [17] and considerably fewer child health specialists, scaling up of HIV treatment in the public health sector relied on task shifting wherein nurses rather than medical doctors, prescribe ART and manage HIV positive children and adults. However, neurodevelopmental assessments are beyond the scope of practice for many nurse clinicians as well as non-pediatric trained medical doctors in this setting.

The aims of this study were 1) to determine whether the TQ screen can identify HIV positive children who *are* and *are not* at risk for neurodevelopmental disability (serious and mild) in order to efficiently refer those who are at risk for further assessment and 2) to characterize neurodevelopmental disabilities in a population based sample of South African pre-school children, including both HIV positive and HIV negative.

## Methods

In order to assess the utility of the TQ as a screen for HIV+ children about to enter school, we compared the caregiver responses on the TQ screen with a disability assessment obtained by a medical doctor in a population sample of 4-6-year-old HIV positive and HIV negative children who were enrolled in the Asenze Study. The Asenze Study is a longitudinal epidemiologic study of health and psychosocial need and related contextual factors among young children. The primary caregiver of all 4-6-year-old children was identified through a door-to-door household survey of five isiZulu tribal areas in the eThekwini District, KwaZulu-Natal, South Africa, and were invited to enrol themselves and the child in a longitudinal study of their health and psychosocial functioning. The area comprises peri-urban dwellings, and is characterized by high levels of HIV infection, food insecurity and unemployment.

Children aged between 4–6 years at the time of the first wave of data collection who were residents in the area for past 6 months were eligible to participate in the study. At the household visit, following informed consent from primary caregivers, the TQ screen for child disability was administered. The TQ is a brief questionnaire for childhood disability designed to be used in a range of cultures in low- and middle-income countries [18]. The child’s caregiver was asked a number of questions to compare the child to peers in the community. The TQ screen had been translated and back translated into isiZulu following standard procedures for psychometric instruments [19]. A child scores positive for a possible disability if one or more of the ten questions are positive. Socio-demographic information was collected for the household, for the index child’s biological parent and/or primary caregiver. Each index child and the child’s primary caregiver, who was responsible for the child’s care and well-being on a regular basis, were then invited for assessments at the Asenze study center. A medical doctor, blinded to the results of the TQ screen, administered a standardized semi-structured pre-coded medical history and physical exam including a developmental history and brief structured observations of functioning in language, motor skills, following instructions adapted from Durkin et al. [20] in order to determine the presence and severity of a neurodevelopmental disability (mild, moderate, severe) in the domains of gross and fine motor function, cognition, speech/language, hearing and vision. Besides the routine clinical history and physical examination by the doctor, children underwent a hearing screen using Distortion Product Otoacoustic Emissions (DPOAE). Those who failed the DPOAE also had tympanography by emittance audiometry. Vision screening, which was performed using a “tumbling E” Snellen eye chart [21].

All adults and children attending the assessment were offered HIV testing with counseling. Consent for HIV testing was obtained by trained counselors in accordance with national guidelines [22].

The caregiver responses to the TQ screen were compared with disability assessments obtained by a medical doctor using a standardized pre-coded history and physical, including hearing and vision testing. These measures form the diagnostic criteria for disability related to gross and fine motor, cognition, speech/language, and vision and hearing. Additional independent assessments of language delay (the Reynell) and cognitive ability (the Grover Counter test) [23]were obtained independent of the doctor by native isiZulu speaking trained mid-level psychological assessors trained by an experienced child psychologist. The Grover Counter test has been developed and validated for the South African population [24, 25].

Comparisons made between HIV positive and HIV negative children were conducted using independent sample t-tests for continuous variables and chi-square tests for categorical variables. Odds ratios and confidence intervals were calculated to assess strength of association. The *sensitivity*, *specificity*, and predictive value (positive and negative) of the TQ were determined for HIV positive and HIV negative subjects. P-values of <0.05 were considered statistically significant.

The Asenze study received ethical approval from the Biomedical Research Ethics Committee of the University of KwaZulu-Natal and from the Institutional Review Board of Columbia University and permission from the local health committee. Children with any health issues, including HIV or a probable disability, were referred to local services for care, as described elsewhere [26].

## Results

**Fig 1:**
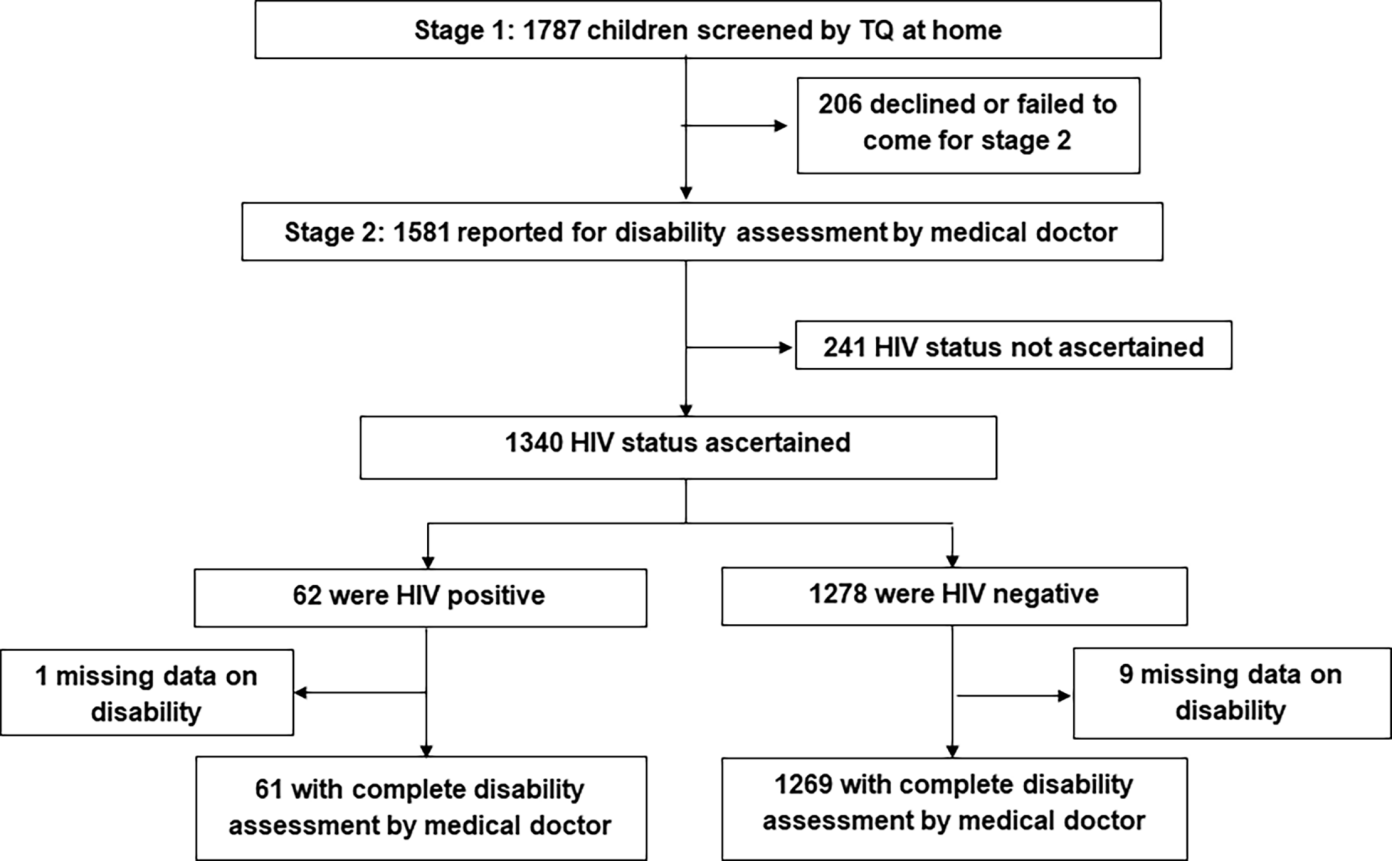
Flow chart enrollment of participants.

The door-to-door survey identified 14,425 households and 2,049 children ages 4–6 years old who were eligible for the study. During the household assessment, 1,787 children were screened with the TQ in their homes during Stage 1 and among these, 1581 reported to the study center for Stage 2 assessment (Fig 1). During the assessment, 1340 (84.8%) children had their HIV status ascertained, either through testing or caregiver report of previous testing, resulting in 62 who were identified by the end of the study as HIV positive (4.6%). Of note, only 20 children were previously known to be HIV positive (less than a third) and 18 of these were receiving ART. Among children who underwent HIV testing, 1330 also completed the standardized disabilities assessment by the medical doctor including 61 of the 62 HIV positive children and 1269 children who tested negative for HIV.

Characteristics of the 1330 children with information on their HIV status and disability are presented in Table 1. Nearly half (45.9%) of HIV positive children were not attending a school or an early childhood educational program compared to about a third (34.8%) of HIV negative children. None of the differences between HIV positive and HIV negative children with respect to age, school enrollment or caregiver arrangement achieved statistical significance. The median previous CD4 count was 190 cells/ml^3^ however results were available for only 16 of the 61 HIV positive children included in this analysis.

**Table 1:** Characteristics of subjects (N = 1330).

Child Characteristics	HIV Positive N = 61	HIV Negative N = 1269	t	P—value
	mean (SD)	mean (SD)		
**Age (years)**	4.9 (0.6)	5.0 (0.6)	0.1	0.96
Previous CD4 count (no/mL)* median (IQR)	190 (384)	-	-	-
	**N (%)**	**N (%)**	**Χ**^**2**^	**P—value**
**Gender, Male**	33 (54.1)	627 (49.4)	0.5	0.47
**School**			3.1	0.21
Not attending	28 (45.9)	442 (34.8)		
Early childhood development program or Crèche (nursery school)	12 (19.7)	312 (24.6)		
Primary school	21 (34.4)	515 (40.6)		
**On Antiretroviral therapy**	18 (31.0)	-	-	
**Caregiver**			0.0	0.99
Mother	43 (70.5)	906 (71.4)		
Grandmother	10 (16.4)	202 (15.9)		
Other Relative/Unknown	8 (13.1)	161 (12.7)		

* CD4 refers to laboratory test that measures the number of CD4 T lymphocytes (CD4 cells) in a sample of blood. In people with HIV, the CD4 count is the most important laboratory indicator of immune function and the strongest predictor of HIV progression. Previous CD4 count was only available for 16 HIV positive children.

The characteristics of the adult caregivers (n = 1231) of study subjects are presented in Table 2. Over 94% (1,164) of the adult caregivers either agreed to a voluntary HIV test or had been tested previously and reported their HIV status. Ninety–six adults were caring for >1 child enrolled in the study so Table 2 describes the 1231 adults who participated in the study. More than two thirds (70.5%) of the caregivers of HIV positive children were HIV positive themselves compared to 26.1% of the caregivers of HIV negative children. A third (33.3%) of mothers of HIV positive children were either dead, not living with the child or had abandoned the child compared to 19.9%of the mothers of HIV negative children. The high HIV prevalence among participating caregivers is in line with the HIV prevalence reported in KwaZulu-Natal province at the time that the study was conducted [15, 27].

**Table 2:** Characteristics of children’s caregivers (n = 1231).

Caregiver’s Characteristics	HIV Positive N = 57	HIV Negative N = 1174	t	P—value
	mean (SD)	mean (SD)		
***Age (years)***	36.1 (12.2)	35.0 (12.2)	0.7	0.49
	**N (%)**	**N (%)**	**Χ**^**2**^	**P–value**
***Caregiver’s HIV Status***			61.7	<0.001
Negative	13 (22.8)	807 (68.7)		
Positive	39 (68.4)	305 (26.0)		
Refused Consent/Consent Pending	5 (8.8)	62 (5.3)		
***Mothers Whereabouts***			12.4	<0.01
Living with child	38 (66.7)	940 (80.1)		
Not living with child	5 (8.8)	125 (10.7)		
Abandoned family/Unknown	6 (10.5)	39 (3.3)		
Dead	8 (14.0)	70 (6.0)		
***Mother’s Education***			3.4	0.58
Primary or no Education	9 (15.84)	159 (13.52)		
≥ Secondary	40 (70.2)	892 (76.0)		
Unknown	8 (14.0)	123 (10.5)		

Table 3 presents a comparison of disability status as determined by the TQ screen stratified by HIV status. Scores were not summarized for individuals with missing information on at least one item. Nearly half of the children (45.5%) screened positive on at least one of the TQ items. Overall, HIV positive children were more likely to screen positive on at least one TQ item than HIV-negative children (59.3 vs 42.7%, p = 0.01). Among 61 HIV positive children 24 (40.7%) did not screen positive on a single TQ question that was indicative of a disability, 15 (25.4%) screened positive on one TQ question, 10 (17.0%) screened positive on two TQ questions, and 10 (17.0%) screened positive on three or more TQ questions. Whereas among HIV-negative children 701 (57.2%) did not screen positive on a single TQ question that was indicative of a disability, 327 (26.7%) screened positive on one TQ question, 99 (8.1%) screened positive on two TQ questions, and 97 (7.9%) screened positive on three or more TQ questions. Caregivers of HIV positive children were nearly 4 times more likely to report that their child was delayed in sitting, standing or walking (OR 3.9, 95% CI = 2.1–7.2) and nearly 3 times more likely to report that their child had difficulty walking or had weakness in the arms or legs (OR = 2.7, 95%CI = 0.8–9.34) comrepared to HIV-negative children.

**Table 3:** TQ screening results: Caregiver’s positive responses regarding the child.

Ten Questions	HIV positive N = 61	HIV Negative N = 1269	Odds Ratio 95% CI*
	N (%)	N (%)	
1. Compared with other children, did (name) have any serious delay in sitting, standing, or walking?	15 (24.6)	98 (7.7)	**3.9 (2.1–7.2)**
2. Compared with other children, does (name) have difficulty seeing?	6 (9.8)	102 (8.1)	1.2 (0.5–3.0)
3. Does (name) have difficulty hearing?	15 (24.6)	184 (14.6)	**1.9 (1.1–3.5)**
4. Does (name) have difficulty understanding what you are saying?	9 (14.8)	99 (7.5)	2.0 (1.0.– 4.3)
5. Does (name) have difficulty in walking or moving arms or weakness?	3 (4.9)	23 (1.8)	2.8 (0.8–9.6)
6. Does (name) sometimes have fits, become rigid or lose consciousness?	6 (9.8)	66 (5.2)	2.0 (0.8–4.8)
7. Does (name) learn to do things like other children the same age? (recorded if answer is no)	8 (13.1)	77 (6.1)	**2.3 (1.1–5.1)**
8. Does (name) have difficulty making himself understood in words?	6 (10.0)	71 (5.6)	1.9 (0.8–4.5)
9. Is (name)’s speech in any way different from normal?	7 (11.7)	163 (13.2)	0.9 (0.4–2.0)
10. Compared with other children of his age, does (name) appear in any way mentally slow, delayed or behind?	8 (13.1)	08 (6.3)	**2.2 (1.0–4.9)**
Ten Questions Summary**			
No answers indicating possible disability	24 (40.7)	701 (57.2)	**0.5 (0.3–0.9)**
At least one answer indicating possible disability	35 (59.3)	523 (42.8)	**2.0 (1.2–3.3)**
One answer indicating a possible disability	15 (25.4)	327 (26.7)	0.9 (0.5–1.7)
Two answers indicating a possible disability	10 (17.0)	99 (8.1)	**2.3 (1.1–4.7)**
Three or more answers indicating possible disability	10 (17.0)	97 (7.9)	**2.4 (1.2–4.8)**

* Bold ORs indicate statistically significant results at p < .05

** Scores were not summarized for individuals with missing information on at least one item

Table 4 presents a comparison of disability status as determined by the medical doctor’s assessment (serious, mild and no disability) stratified by HIV status. Children with uncertain status were combined with the other disability groups because the medical doctor was unable to complete the disability assessment for a number of domains as a result of the child being aggressive, uncooperative or shy. In a few instances, a child had disability in another domain, which might have interfered with the doctor's ability to complete the respective assessment. Uncertain diagnoses occurred in <3% of medical assessments for all types of disability. Receiving an uncertain diagnosis occurred among a higher proportion of HIV positive children than HIV negative children (data available from authors). Compared to HIV negative children, HIV positive children were more likely to be diagnosed by the doctor with gross motor disability (OR = 3.5, 95%CI = 1.3–9.2) and hearing disability (OR = 2.5, 95%CI = 1.2–5.3). Table 4 also presents a comparison of cognition and language delay as determined by the Grover Counter test and the Reynell, respectively, which were conducted independently of the medical assessment by mid-level psychological assessors trained by an experienced child psychologist -level, stratified by HIV status. Compared to HIV negative children, HIV positive children were also more likely to be diagnosed with cognitive delay (OR = 2.2, 95%CI = 1.2–3.9) and language delay (OR = 4.3, 95%CI = 2.2–8.4)

**Table 4:** Types of disability assessment comparing any risk of disability (mild/moderate/severe/uncertain) to no disability by HIV status.

Types of Disability	Total sample n = 1330	HIV positive N = 61	HIV Negative N = 1269	Odds Ratio (95% CI)
		N (%)	N (%)	
***Medical Doctors Assessment***				
***Gross Motor***				
None	1293 (97.2)	56 (91.8)	1237 (97.5)	1
Any risk of disability	37 (2.8)	5 (8.2)	32 (2.5)	**3.45 (1.30–9.19)**
***Fine Motor***				
None	1162 (87.4)	51 (83.6)	1111 (87.6)	1
Any risk of disability	168 (12.6)	10 (16.4)	158 (12.4)	1.38 (0.69–2.77)
***Hearing***				
None	1239 (93.2)	52 (85.3)	1187 (93.5)	1
Any risk of disability	91 (6.8)	9 (14.7)	82 (6.5)	**2.51 (1.19–5.26)**
***Vision***				
None	1201 (90.3)	57 (93.4)	1144 (90.2)	1
Any risk of disability	129 (9.7)	4 (6.6)	125 (8.8)	0.64 (0.23–1.80)
***Speech***				
None	1272 (95.6)	57 (93.4)	1215 (95.7)	1
Any risk of disability	58 (4.4)	4 (6.6)	54 (4.3)	1.58 (0.55–4.51)
***Cognition***				
None	1279 (96.2)	57 (93.4)	1222 (96.3)	1
Any risk of disability	51 (3.8)	4 (6.6)	47 (3.7)	1.82 (0.64–5.24)
***Psychological tests***				
***Grover***				
None	1069 (82.6)	41 (69.5)	1028 (83.2)	1
Cognitive delay	226 (17.4)	18 (30.5)	208 (16.8)	**2.17 (1.22–3.85)**
***Reynell***				
None	1243 (93.8)	48 (80.0)	1195 (94.5)	1
Language delay	82 (6.2)	12 (20.0)	70 (5.5)	**4.27 (2.17–8.40)**

Table 5 presents the results of disability status as determined by the medical doctor’s assessment (serious, mild and no disability) stratified by positive vs. negative TQ screening results (i.e. at least one positive answer indicating possible disability vs. none). TQ screening results were collapsed in this way because the TQ was developed to identify serious disability and it has been shown to be a valid screening tool for identifying the presence of disability (not necessarily specific types of disability) [11–13, 18, 20]. Children where the doctor was uncertain whether there was a disability were excluded from this analysis. The results in Table 5 can be used to calculate the sensitivity, specificity, negative and positive predictive value of the TQ for detection of mild disability vs. no disability. Thirteen (22.0%) HIV positive children and 230 (18.5%) HIV-negative children were identified by the medical assessment as having a mild disability. Two HIV positive children with negative TQ screens were determined by the medical doctor to have a mild disability, including 1 child with abnormal vision and 1 child with abnormal vision and delayed fine motor skills. Among HIV positive children, the sensitivity and specificity of the TQ for mild disability was 84.6% and 51.2%, respectively. The positive predictive value was 34.4% and the negative predictive value was 91.7%. Among HIV negative children, the sensitivity and specificity of the TQ for mild disability was 63.9% and 63.9%, respectively. The positive predictive value was 29.6% and the negative predictive value was 88.2%. Results presented in Table 5 can also be used to calculate the sensitivity of the TQ for detection of serious (severe/moderate) vs. no disability. Three (5.1%) HIV positive children and 41 (3.3%) HIV-negative children were identified by the medical assessment as having a serious disability. These numbers were used to calculate sensitivity, specificity, negative and positive predictive value of the TQ for detecting serious disability vs. no disability. Among 24 HIV positive children with a positive TQ screen (and either serious or no disability), 21 were found at medical assessment to have no disability. There were no HIV positive children with a negative TQ result that were determined to have a serious disability (i.e. there were no false negatives). Therefore, among HIV-positive children, the sensitivity and specificity of the TQ for serious disability was 100.0% and 51.2%, respectively. The positive predictive value was 12.5% and the negative predictive value was 100.0%. Among 387 HIV-negative children with a positive TQ screen (and either serious or no disability), 350 were found at medical assessment to have no disability. There were 4 HIV negative children with a negative TQ screen who were found at medical assessment to have a serious disability (i.e. there were 4 false negatives). Therefore, among HIV-negative children, the sensitivity and specificity of the TQ for serious disability was 90.2% and 63.9%, respectively. The positive predictive value was 9.6% and the negative predictive value was 99.4%.

**Table 5:** Results of medical doctor disability assessment for children screened by TQ.

Medical doctor’s assessment of disability* –HIV positive				
**TQ screening**	**Serious disability**	**Mild disability**	**No disability**	**Total**
	**N (%)**	**N (%)**	**N (%)**	
Positive	3 (8.6)	11 (31.4)	21 (60.0)	35
Negative	0 (0.0)	2 (8.3)	22 (91.7)	24
	**%**	**%**		
**Sensitivity**	100.0%	84.6%	Reference	
**Specificity**	42.9%	51.2%	Reference	
**Positive predictive value**	12.5%	34.4%	Reference	
**Negative predictive value**	100.0%	91.7%	Reference	
Medical doctor’s assessment of disability* –HIV negative				
**TQ screening**	**Serious disability**	**Mild disability**	**No disability**	**Total**
	**N (%)**	**N (%)**	**N (%)**	
Positive	37 (6.9)	147 (27.5)	350 (65.5)	534
Negative	4 (0.6)	83 (11.7)	620 (87.7)	707
	**%**	**%**		
**Sensitivity**	90.2%	63.9%	Reference	
**Specificity**	58.6%	63.9%	Reference	
**Positive predictive value**	9.6%	29.6%	Reference	
**Negative predictive value**	99.4%	88.2%	Reference	

* Children where the doctor was uncertain whether there was a disability were excluded from this analysis.

## Discussion

In this first report of the use of the TQ screen to validate its use in HIV-positive children in a low resource setting, a high percentage of children screened positive for disability. We identified a high yield (4.6%) of HIV positive children through a door-to-door household survey, more than a third of whom were previously undiagnosed [28]. There were elevated levels of neurodisability overall and more children with several types of disability among HIV-positive children. The findings of the medical doctor were largely corroborated by structured tests by the independent mid-level assessors. Overall, the TQ was found to have high sensitivity for detecting serious developmental disabilities in children with and without HIV. However, the low specificity and positive predictive values make it important to consider whether screening with the TQ would be an efficient use of resources.

This is also the first report of the use of the TQ screen in the isiZulu language. The proportion who screened positive among both HIV positive and HIV negative children in this study are among the highest reported in population-based studies. Gross motor concerns were especially prominent including: delays in learning to sit and stand, difficulty walking or moving arms, and weakness. Although data are limited, the proportion of TQ-assessed, disability in HIV-negative children in this study is comparable to those from other sub-Saharan African countries including Central African Republic (48%) but higher than those reported from Cameroon (33%) [12]. Gross motor problems were especially prominent, including delays in learning to sit and stand, difficulty walking or weakness in the arms or legs. Overall, HIV positive children were more likely to screen positive on any of the TQ items than HIV-negative children, similar to findings of a recent clinic-based study from Malawi that included HIV positive children ages 2–9 years and HIV negative siblings [29]. However, the proportion of Malawian children who screened positive on TQ (33% of HIV positive and 7% of control children) was lower than proportions we observed. The reason for these differences is not known nor can comparisons be made of the rate and types of underlying disabilities, as no additional assessment beyond TQ screening were conducted in the Malawi study [29].

We observed a higher proportion of disability among HIV positive children, as detected by the TQ screen. Similarly, a higher proportion of HIV positive children had disability in either motor function, vision or hearing, as detected by the medical doctor’s assessment. Hearing and speech disability, while relatively high in general, was also more prevalent among children with HIV, as assessed by the TQ screen, the medical doctor assessment and OAE and Tympanometry in the case of impaired hearing. Our findings of high prevalence of disability among HIV positive children are comparable to other published reports from sub-Saharan Africa. For example, the prevalence of disabilities that we observed are comparable to a population-based study among children in a Tsonga-speaking area of South Africa that also used the TQ screen, which reported a 3.5% prevalence of intellectual disabilities [30]. Differences between what we observed and other studies on developmental disability conducted in sub-Saharan Africa are likely due to differences among the samples in terms of age, sociodemographic characteristics and other determinants, as well as differences in terms of how disability was assessed. For example, motor delay is considerably lower in the present study (as determined by medical assessment) than results reported among 30–72 month old HIV positive children from Kinshasa [31]. Differences in this case are likely due to the inclusion of slightly younger children in the Kinshasa study and their use of a different measure (the Peabody Developmental motor scales). Higher rates of mental and psychomotor developmental delay were reported in a cohort of children less than 18 months old who were born to HIV-positive mothers in Tanzania [32]. Apart from being considerably younger, these children were evaluated by the Bailey Scales of Infant Assessment Experience.

An important finding in this study is the high sensitivity of the TQ in relation to a structured medical doctor assessment together with hearing and vision testing, especially among HIV positive children. The sensitivity that we observed is in keeping within the range generally observed in other studies[20], and is considered with the acceptable range for a developmental screen (80–100%). Similar to these previous findings that the TQ is acceptable as a low-cost and rapid screen for serious disabilities in developing settings, we observed a low positive predictive value among HIV positive children that was within the range of what has been previously observed (3–15%) [20]. This further confirms that the value of the TQ for identifying disability in underserved populations is limited to that of a screen and that more thorough evaluations of children that screen positive are necessary in order to determine whether they truly have a disability and to identify the nature of the disability if present. Also, as noted in some but not all prior studies, the TQ was sensitive for detecting mild disabilities among HIV positive children. Furthermore, these findings are strengthened by the independent use of the Reynell and Grover Counter tests, which detected similar levels of increased likelihood of language delay and cognitive delay among HIV-positive children compared to HIV-negative children. The Reynell and Grover Counter tests are validated tests conducted in isiZulu by mid-level psychological assessors that were trained by an experienced child psychologist and represent more than clinical judgment. This provides some validation of the findings achieved by the medical doctor assessment.

This study has certain limitations. First, because it was population based sample of preschool children, the study only included a small number of HIV positive children across a limited age range. Further evaluation of performance and programmatic utility of the TQ across a wider age and clinical spectrum is warranted. Also, the medical doctor was unable to fully assess a number of children, including many with HIV, due to aggressive, uncooperative or shy behavior. This resulted in an “uncertain” determination of particular disabilities (e.g. gross motor or hearing, see Table 5). It is important to note that this assessment took place as part of a full medical examination. Since the TQ did not include questions on child behavior, it would not identify behavioral difficulties, noted by the doctor which restricted his ability to assess other neurodevelopmental domains. This is a limitation of the instrument and may reflect an underlying area of developmental difficulty which could benefit from referral to specialist assessment targeting behavioral disturbances. Therefore, this gap likely resulted in an underestimation of disability, particularly among those with HIV.

In summary, there are over 3 million children living with HIV. In countries hit hard by the HIV epidemic and where efforts to eliminate maternal to child transmission of HIV are incomplete, sizeable numbers of HIV positive children lack access to ART. This study demonstrates that many HIV positive children have unrecognized neurodevelopmental disabilities and need a wide range of interventions and support to survive and develop. Increased attention to early HIV diagnosis and intervention is necessary to prevent these neurocognitive issues, as far as possible. The ability to rapidly identify children with undiagnosed HIV who need further assessment, intervention or support in health or educational settings would also help to improve outcomes during critical periods of childhood development. The performance of the TQ in this sample indicates utility for screening of HIV positive children who warrant further evaluation for serious neurodevelopmental disability. Despite the TQ’s low specificity and its low positive predictive value among HIV+ children, it still warrants consideration for whether it is the best use of scarce resources. Researchers, public health professionals, and policy makers should consider implementation of this simple screening instrument as it has the potential to greatly reduce the number of children requiring more intensive neurodevelopmental assessment and maximize the use of limited resources in settings with few skilled health care professionals.
